# Effect of the *MySweetheart* randomized controlled trial on birth, anthropometric and psychobehavioral outcomes in offspring of women with GDM

**DOI:** 10.3389/fendo.2023.1148426

**Published:** 2023-06-07

**Authors:** Leah Gilbert, Dan Yedu Quansah, Amar Arhab, Sybille Schenk, Justine Gross, Stefano Lanzi, Bobby Stuijfzand, Alain Lacroix, Antje Horsch, Jardena J. Puder, MySweetheart Research group

**Affiliations:** ^1^ Nepean Clinical School, Faculty of Medicine and Health, The University of Sydney, Sydney, NSW, Australia; ^2^ Obstetric Service, Department Woman-Mother-Child, Interdisciplinary GDM Group Lausanne, Lausanne University Hospital, Lausanne, Switzerland; ^3^ Service d’angiologie, Département Cœur-Vaisseaux, Centre Hospitalier Universitaire Vaudois (CHUV), Lausanne, Switzerland; ^4^ Institute of Higher Education and Research in Healthcare (IUFRS), University of Lausanne, Lausanne, Switzerland; ^5^ Neonatology Service, Department Woman-Mother-Child, Lausanne University Hospital, Lausanne, Switzerland

**Keywords:** sleep, temperament, weight, fat mass, skinfold, prematurity, hypoglycemia, BMI - body mass index

## Abstract

**Introduction:**

Gestational diabetes mellitus (GDM) may negatively affect offspring outcomes. A lifestyle intervention may therefore not only improve maternal, but also offspring outcomes. The effects of lifestyle interventions on birth, anthropometric, and psychobehavioral outcomes in offspring of women with GDM need further evidence.

**Design:**

The *MySweetheart trial* is a monocentric single-blind randomized controlled trial in 211 women with GDM. It tested the effect of a pre- and postpartum multidimensional interdisciplinary lifestyle and psychosocial intervention focusing on both the mothers and their infants and its effects on maternal (primary outcomes) and offspring (secondary outcomes) metabolic and psychobehavioral outcomes compared with guidelines-based usual-care. This paper focuses on offspring’s birth, anthropometric, and maternal report of psychobehavioral outcomes at singular timepoints.

**Methods:**

Women with GDM aged ≥18 years, between 24-32 weeks of gestation, speaking French or English were included and randomly allocated to either the intervention or to an active guidelines-based usual-care group using a 1:1 allocation ratio. The intervention lasted from pregnancy until 1 year postpartum and focused on improving diet, physical activity, and mental health in the mother. For the offspring it focused on supporting breastfeeding, delaying the timing of introduction of solid foods, reducing the consumption of sweetened beverages, increasing physical activity of the family, and improving parental responsiveness to infant distress, hunger, satiety and sleeping cues, and difficult behavior.

**Results:**

Adverse birth and neonatal outcomes rarely occurred overall. There were no differences between groups in offspring birth, neonatal, anthropometric, or psychobehavioral outcomes up to one year. After adjustments for maternal age and the offspring’s sex and age, there was a borderline significant between-group difference in birth length (*β*:-0.64, *CI*:-1.27; -0.01, *p*: 0.05), i.e., offspring of mothers in the intervention group were born 0.64 cm shorter compared to those in the usual-care group.

**Conclusion:**

This is the first pre- and postpartum multidimensional interdisciplinary lifestyle and psychosocial intervention in GDM focusing on both the mother and the offspring. It did not lead to a significant improvement in most birth, anthropometric, and psychobehavioral outcomes in offspring of women with GDM. ClinicalTrials.gov Identifier: NCT02890693

## 1 Introduction

Gestational diabetes mellitus (GDM) is a glucose intolerance first diagnosed during pregnancy that does not fulfil the criteria for pre-existing diabetes (1). The prevalence of GDM in Switzerland is 10.8% (2). GDM is associated with adverse offspring birth and neonatal outcomes, such as perinatal mortality, preterm delivery, and caesarean delivery (3, 4). GDM is also associated with the offspring’s anthropometry, as it leads to a higher risk of macrosomia, high birth weight, large for gestational age (LGA), accelerated weight gain after delivery, and increased risk of obesity and diabetes later in life (5–8). Breastfeeding has been associated with lower infant fat mass (9) and lower risk of obesity and better overall health in the general population (10). However, women with GDM are at greater risk of delayed breastfeeding initiation and low milk supply (11). Less social support for breastfeeding has been reported among mothers with GDM, which may partly contribute to these trends (12). Additionally, GDM-affected pregnancies carry a higher risk of obesity, cesarean delivery and neonatal hypoglycemia, which may delay breastfeeding initiation after birth (12). Furthermore, the positive effect of breastfeeding on lower fat mass has not been demonstrated in the offspring of mothers with GDM (13, 14).

GDM is also associated with worsened maternal mental health, particularly with a higher risk of maternal depression (15–17). This may also indirectly affect the offspring’s psychobehavioral outcomes. Indeed, maternal depression in women with GDM is associated with shorter sleep duration in the offspring at one year of age, which may in turn negatively impact the offspring’s development (18). In women with obesity, maternal perception of offspring difficult behavior is also more frequent and is associated with poorer offspring development, such as low mood and inadaptability up to 12 years of age; however, this still needs to be investigated in GDM populations (19, 20). In prior studies, GDM was associated with a higher risk of hyperactivity disorder in children aged six, but there is no evidence of GDM affecting earlier behavior (21). Given the risks associated with GDM, it is of utmost importance to intervene in pregnancy and the postpartum period.

Prior to the conception of the *MySweetheart trial*, some lifestyle intervention studies mainly focusing on diet and physical activity in the mother had investigated the effect of these interventions on adverse birth and neonatal outcomes in offspring of mothers with GDM and mainly show there is low evidence for improvements in these outcomes for the intervention group (22). Another study also investigated the impact of medication in women with GDM on the anthropometric and metabolic outcomes of children (7-9 years old) (23). Nonetheless, no prior studies in women with GDM have investigated the impact of a psychosocial and lifestyle intervention, containing an intervention conducted in both the mother and the offspring on the offspring’s birth, neonatal, anthropometric and psychobehavioral outcomes. In the general population, modifiable risk factors of adverse birth and neonatal outcomes, such as preterm delivery, include reinforcing social support and improving nutrition in mothers during pregnancy (24, 25). With regards to improvements in the offspring’s anthropometry, a study showed that intervening exclusively on maternal lifestyle behavior, including nutrition, did not favorably influence offspring adiposity outcomes up to five years of age (26). Furthermore, previous studies performed in different populations, containing interventions focusing on breastfeeding (versus formula feeding), timing of solid food introduction, sweetened beverage consumption, parental responsiveness to feeding cues and offspring distress, and maternal and offspring physical activity have shown effects on the offspring’s anthropometry (27–32). These postnatal interventions could also improve similar outcomes in offspring of women with GDM. Guidelines provided by the World Health Organization (WHO) at the time of the production of the protocol encouraged women to breastfeed exclusively for at least six months (33). European and Swiss Guidelines for the introduction of solid foods recommended that it should be initiated after at least four months of age to reduce the risk of obesity later in life (34–37). Prior parenting skills interventions have focused on anticipatory guidance, offspring cues, and distress to positively influence self-regulatory capacities to reduce the offspring’s obesity risk (31, 38, 39). One intervention study in the general population focusing on responsive parenting to feeding cues showed that when parents used responsive feeding practices, their offspring gained weight more slowly than the controls and were less likely to be overweight at 12 months (40). Responsive feeding by parents may also be an efficient strategy in ensuring healthy growth of the offspring and guidelines on how to effectively use these behaviors have been proposed in recent years (41, 42). Regarding physical activity, it is recommended that offspring under one year of age should be physically active several times a day through interactive floor-based play, as it improves measures of adiposity, motor skill development, and cognitive development (43). Finally, different parental interventions in the general population have shown positive effects on the offspring psychobehavioral outcomes, such as improvements in sleep, socioemotional development, and behavioral problems (44, 45).

Previous studies in GDM investigating the impact of maternal lifestyle interventions on offspring health outcomes are scarce. Importantly, there are no published intervention trials intervening in the offspring of mothers with GDM (1). The *MySweetheart trial* is an interdisciplinary randomized-controlled trial in mothers with GDM. It is the first pre-and postpartum complex intervention that tested the effect of a multidimensional lifestyle and psychosocial trial intervening on both the mother and their offspring on maternal (primary outcomes) and offspring (secondary outcomes) metabolic and psychobehavioral outcomes.

The aim of this paper was to focus on the secondary outcomes of the trial, i.e., on differences in the offspring’s birth, anthropometric or psychobehavioral outcomes up to one year of age between the intervention and an active guidelines-based usual-care group.

## 2 Methods

### 2.1 Trial design

The *MySweetheart trial* (RCT: ClinicalTrials.gov Identifier: NCT02890693) was a monocentric single-blind randomized controlled trial (RCT) that tested the effect of a pre- and postpartum multidimensional interdisciplinary lifestyle and psychosocial intervention on metabolic and psychological outcomes in mothers with GDM and their offspring compared to an active guidelines-based control group. More details are provided in the study protocol (46). The primary aims of the trial were to improve maternal mental and metabolic health outcomes. Secondary aims were to improve offspring birth, anthropometric, and psychobehavioral outcomes. The study protocol was approved by the Human Research Ethics Committee of the Canton de Vaud (study number 2016-00745).

### 2.2 Participants

Women aged ≥18 years, diagnosed with GDM according to the IADPSG criteria (47), between 24-32 weeks gestational age (GA), and who understood French or English were included. We excluded women on strict bed rest, with pre-existing diabetes or if they had a current severe mental health disorder which included the presence of a current psychotic episode or acute suicidal risk. We recruited women from the diabetes and pregnancy clinic of the Lausanne University Hospital (CHUV) or women that were referred from other antenatal care clinics or obstetricians in private practices. The first patient recruitment started on September 2, 2016, and the last patient one year follow-up visit was October 25, 2021. During the COVID-19 lockdown, we suspended recruitment, testing, and follow-up of participants for three months (until 26.5.2020), and partially for an additional two months (i.e., a total of five months) due to the extension of restriction guidelines in Switzerland. To avoid unforeseen dropouts linked to the second COVID-19 wave, we recruited 11 more patients. Besides this, there were no changes to the protocol after trial commencement (46). Mothers taking part in the study provided their written informed consent for their and their child’s participation in this study.

### 2.3 Active lifestyle and guidelines-based usual-care group

The usual-care was an active lifestyle and guidelines-based clinical control group. All mothers and their offspring were followed-up according to the guidelines of the American Diabetes Association (ADA), the International Association of the Diabetes and Pregnancy Study Groups (IADPSG) and the Endocrine Society (1, 47, 48) and according to the NICE guidelines regarding mental health (49). They were seen at 24-32 weeks GA either by a physician, or a diabetes-specialist nurse, and followed until birth (see Horsch et al., 2018 for more details (46)). Patients received information on GDM, were counseled about lifestyle changes and optimal gestational weight gain (GWG) and were taught how to perform self-control of blood glucose (50). They had one appointment with a registered dietician to promote favorable glucose controls with individualized dietary advice. Mothers were also advised to reduce sedentary behavior and engage in physical activity according to the Endocrine Society guidelines and received general advice for breastfeeding (47). Mothers were followed up at 6-8 weeks and one year postpartum and were given advice about weight loss and lifestyle behaviors at these time-points. No other specific intervention was delivered regarding the offspring (51).

### 2.4 Intervention group

Complex interventions, such as the *MySweetheart trial* are characterized by several interacting components, several outcomes, a high degree of flexibility, and the possibility of tailoring the intervention. The Health Action Process Approach (HAPA) was chosen as the theoretical framework for this behavior change intervention (46). The intervention started after the first clinical visit in the diabetes and pregnancy clinic and lasted up to one year postpartum. The design of the intervention components was based on three informal focus group sessions with patients (both pregnant women with GDM and postpartum women) and on feedback from experienced clinicians. The usual-care and intervention groups of the *MySweetheart trial* have been previously described (46, 52).

Regarding the offspring (secondary aims), mothers received support for breastfeeding, recommendations regarding timing of solid food introduction, consumption of sweetened beverages, parental responsiveness to distress, hunger, satiety and sleeping cues and difficult behavior in the offspring, as well as his/her physical activity needs. Breastfeeding was additionally supported with the help of the midwife at birth and afterwards, as desired (51). Mothers were encouraged to maintain continuous (not necessarily exclusive) breastfeeding up to six months postpartum and were informed about its health benefits at the first interdisciplinary visit between 6-8 weeks postpartum (51). The first additional interdisciplinary visit at 4 months postpartum focused on maternal and offspring diet, breastfeeding, and introduction of solid foods, which was encouraged to take place no earlier than at four months of age, in accordance with the European Society for Paediatric Gastroenterology, Hepathology and Nutrition and Swiss guidelines at the time of protocol production (36, 37). During the second interdisciplinary visit at seven months postpartum, parental regulation of offspring distress or difficulty and self-regulation capacity were reinforced through parental education with the help of a psychologist and the lifestyle coach. This theme, particularly how to recognize and react to hunger and satiety cues and how to soothe babies when in distress, was already discussed in the peer support group workshop during pregnancy (43). During the last interdisciplinary visit at 10 months postpartum and during the second peer support group workshop in the postpartum, mothers were encouraged to increase their offspring’s physical activity (target 180 min/day) and to reduce sedentary behavior, with a particular focus on screen time (43). The peer-support group in the postpartum focused on offspring physical activity, while both encouraged peer exchanges and used different tools and brochures (53, 54)

Following these visits, the lifestyle coach followed up with the goals by summarizing them through a text message sent to the mother. Questions and concerns were discussed during the bimonthly phone calls. Furthermore, co-parents were also invited to each session to reinforce a unified approach for both parents regarding the health goals of their offspring.

### 2.5 Data collection and study visits

At the first visit, maternal anthropometric measures (height (cm), weight (kg)) were measured and pre-pregnancy weight was self-reported. Medical information, sociodemographic variables, and social support of the mother were also collected. HbA1c in pregnancy and the requirement for maternal medical treatment were recorded at the end of the pregnancy. In addition, the type of delivery was collected at the first postpartum visit. Breastfeeding presence (yes/no) was assessed by clinicians and self-reported at 6-8 weeks and 1 year. When clinician report of breastfeeding was missing, we retrieved the information from the participant’s report.

At birth, the offspring’s baseline measures including birth and neonatal outcomes, gestational age, sex, weight, and height were assessed. At 6-8 weeks and at the one-year visits, the offspring’s weight, size, and skinfolds were measured and mothers completed additional self-report questionnaires at all visits. Data collection and outcomes were measured at the diabetes and pregnancy clinic at the Lausanne University Hospital at all time points. Data regarding birth and neonatal outcomes were extracted from medical charts and records. Secondary offspring outcomes also contained cardiometabolic laboratory outcomes in the cord blood of the offspring, but these are not presented here, as we have much fewer infants with this type of data (n=46 of the total 211 subjects) and thus the sample size would have been significantly reduced.

### 2.6 Offspring outcome measures

#### 2.6.1 Adverse birth and neonatal outcomes

Adverse birth and neonatal outcomes including neonatal hospitalization to the neonatal intensive care unit, hyperbilirubinemia (total serum bilirubin levels equal or above 15 mg/dL (257 μmol/L) (55)), hypoglycemia (capillary or venous glucose value ≤2.5 mmol/l (55)), and preterm delivery (<37 GA) were extracted from the participants medical charts and records.

#### 2.6.2 Anthropometric outcomes

Birth weight (g) and length (cm) were recorded at birth using calibrated electronic offspring scales (Seca^®^). Macrosomia was defined as birthweight ≥4000 g. Large-for-gestational-age (LGA) and small-for-gestational-age (SGA) were defined as sex-and GA–specific birth weight >90th and <10th centile, respectively, according to the International Fetal and Newborn Growth Consortium for the 21st Century (INTERGROWTH-21st) guidelines (56). We calculated percentiles and z-scores for length and weight variables at birth according to the Intergrowth 21st newborn size guidelines (56). We measured offspring length (cm) and weight (kg) at 6-8 weeks and at one year and calculated their BMI, and the respective z-scores according to the WHO offspring growth standards anthropometric tool at both time points (57). We used offspring BMI z-scores to classify them as underweight (lower than −2 SD), normal (from −2 SD to < 1 SD), overweight (1 SD to <2 SD) or obese (≥ 2 SD) (58, 59). We measured offspring skinfolds using the Harpenden calipers, on the biceps, the triceps, the subscapular and the iliac crest at 6-8 weeks and one year (60, 61) Each skinfold was measured up to three times at each anatomical site and the mean of all measures was used. Skinfold thickness was calculated as the sum of the mean of the four skinfold measures for the 6-8 postpartum and one-year visits. Total fat mass and fat free mass were estimated from bioimpedance analysis (BIA) (Akern BIA 101) at one year using the formula of Butte et al. (62).

#### 2.6.3 Psychobehavioral outcomes

At one year postpartum, mothers completed two questionnaires assessing psychobehavioral outcomes of their offspring. Firstly, the Difficult Child subscale of the Parenting Stress Index short-form assessed the mother’s perception of offspring’s difficulties with self-regulation with 12 items on a 5-point Likert scale ranging from 1 ‘totally agree’ to 5 ‘totally disagree’ (46, 63). Secondly, the Brief Infant Sleep Questionnaire evaluated offspring’s nighttime sleep duration, the number of night wakings, and whether the mother perceived her offspring’s sleep as a problem was assessed with 3 of the 14 items (64).

### 2.7 Sample size

**Figure 1:**
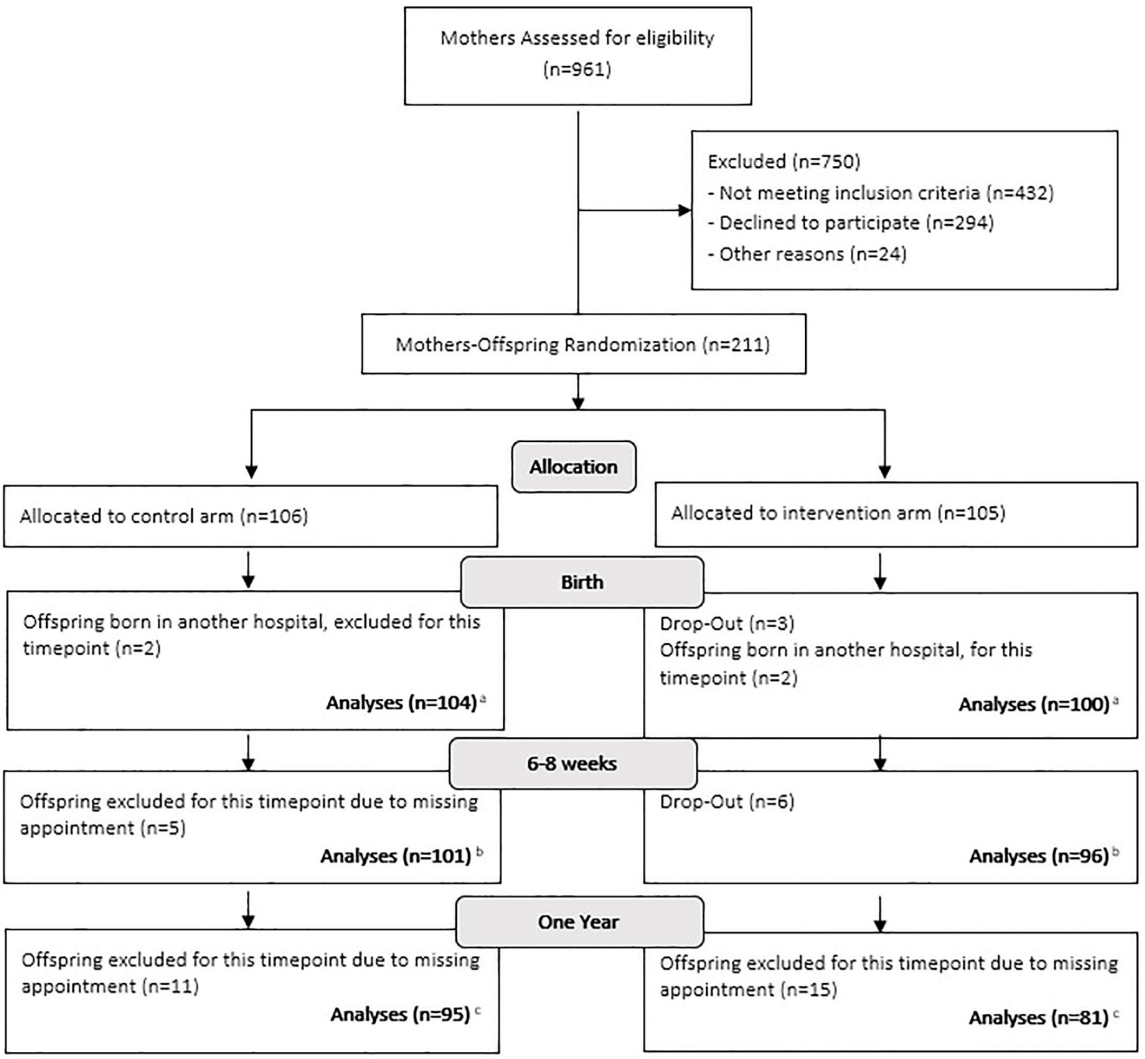
Flow-chart. **(A)** For the outcomes: hospitalization at birth, hyperbilirubinemia and hypoglycaemia, we were able to retrieve information from the other hospitals to analyse the data on 211 individuals. We were also able to retrieve birth length for 4 individuals, thus length analyses was made on 208 individuals. **(B)** For two separate individuals one length and one size was missing, bringing the numbers down from 197 to 196 for weight and length and to 195 for BMI and z-scores. Also, we were unable to conduct skinfolds on 7 individuals. **(C)** One individual did not have his weight and length measured, thus why there are 175 individuals for these measures. We did not measure 13 individuals for their skinfolds and BIA, mostly due to maternal choice which left us with 163 individuals for both measures. Finally, at one year only 153 mothers filled out the Difficult Child Indicator Scale and only 161 mothers filled out the Brief Infant Sleep Questionnaire, in which one mother omitted to answer the total nighttime sleep duration and the number of night wakings.

We estimated the sample size based on the expected differences in primary outcomes of the *MySweetheart trial*, i.e., differences in maternal weight and depression symptoms between the usual-care and intervention groups (see (46)). Of the n=211 included mothers, n=106 mothers and their offspring were randomized to the usual-care group and n=105 to the intervention group. Details on the number of participants per time point can be found in **Figure 1** below and more details on the total number of individuals we have per variable can be found in the first column of **Tables 1** and **2**, and this is also described in the flow-chart legend. For twin pregnancies (n=3) we decided to compare the data of the first twin to all other offspring outcomes and to assess if there were any significant differences. As there were none, we used the data for the first twin in all pregnancies.

**Table 1:** Maternal characteristics.

	*Total N*	All (n=179)	Intervention group (n=85)	Usual-care group (n=95)	Differences between groups *p-value*	
Age (years)	210	33.63 (4.97)	34.48 (5.15)	32.79 (4.65)	**0.01**	
Pre-pregnancy BMI	210	25.90 (5.45)	25.93 (5.42)	25.86 (5.51)	0.93	
BMI at the 1^st^ GDM visit	208	29.68 (5.04)	29.54 (4.87)	29.54 (4.87)	0.69	
Gestational weight gain (kg)	171	12.67 (6.44)	11.79 (6.55)	13.45 (6.27)	0.09	
HbA1c at the end of pregnancy (%)	205	5.11 (0.32)	5.16 (0.33)	5.07 (0.31)	0.054	
Caesarean section (yes)	198	73 (37%)	40 (41%)	33 (33%)	0.25	
Maternal medical treatment requirement (yes)	198	90 (45%)	43 (43%)	47 (48%)	0.48	
Social support (lives with partner) (yes)	211	181 (56%)	94 (90%)	87 (82%)	0.20	
Nulliparous (yes)	211	120 (47%)	57 (54%)	63 (59%)	0.45	
Ethnicity	189				0.59	
Swiss		62 (33%)	32 (35%)	30 (31%)	
European		79 (42%)	35 (38%)	44 (45%)	
Non-European		48 (25%)	25 (27%)	23 (24%)	
Educational level	177				0.98	
Compulsory education not complete		2 (1%)	1 (1%)	1 (1%)		
Compulsory education complete		23 (11%)	12 (11%)	11 (10%)	
Secondary education		19 (9%)	9 (9%)	10 (9%)	
Apprenticeship		42 (20%)	19 (18%)	23 (22%)	
Higher education		91 (43%)	41 (39%)	50 (47%)	
Breastfeeding at 6-8 weeks (yes)	202	195 (97%)	96 (96%)	99 (97%)	0.60	
Breastfeeding at one year (yes)	171	122 (71%)	55 (71%)	67 (72%)	0.64	

Continuous and normally distributed variables were described as means and standard deviations and ordinal outcomes were described as n (%).

p-values were calculated via t-test for numeric outcomes and via Chi2 for ordinal outcomes. The bold p-values represent values <0.05.

BIA, Bioimpedance; BMI, Body Mass Index.

**Table 2:** Offspring characteristics.

		*Total N*	All (n=179)	Intervention group (n=85)	Usual-care group (n=95)	Differences between groups *p-value*	
General information							
Offspring sex (female)		205	97 (47%)	46 (46%)	51 (49%)	0.51	
Offspring GA (weeks)		204	39.33 (1.59)	39.54 (1.33)	39.29 (1.79)	0.06	
Adverse Birth and neonatal outcomes							
Neonatal Hospitalization (yes)		211	10 (5%)	6 (6%)	4 (4%)	0.51	
Hyperbilirubinemia needing treatment (yes)^a^		211	4 (2%)	1 (1%)	3 (3%)	0.32	
Hypoglycaemia (yes)^b^		211	4 (2%)	2 (2%)	2 (2%)	0.99	
Prematurity (<37 GA) (yes)		204	17 (8%)	5 (5%)	12 (12%)	0.09	
Macrosomia (>4000g) (yes)		204	17 (8%)	10 (10%)	7 (7%)	0.40	
Large for gestational age ^c^		204	22 (11%)	10 (10%)	12 (12%)	0.72	
Small for gestational age ^d^		204	23 (11%)	12 (12%)	11 (11%)	0.75	
Anthropometric Outcomes at birth							
Length (cm)		208	49.18 ()	49.03 (2.57)	49.32 (2.77)	0.44	
Weight (kg)		204	3.29 (0.53)	3.29 (0.54)	3.28 (0.52)	0.82	
Weight z-score		204	0.14 (1.05)	0.05 (1.12)	0.22 (0.98)	0.25	
Timing of Assessments at 6-8 weeks							
Offspring age (months)		197	1.41 (0.53)	1.39 (0.51)	1.44 (0.56)	0.51	
Anthropometric Outcomes at 6-8 weeks							
Length (cm)		196	55.77 (2.94)	55.85 (2.69)	55.70 (3.18)	0.73	
Weight (kg)		196	4.72 (0.72)	4.75 (0.67)	4.69 (0.77)	0.56	
BMI (kg/m^2^)		195	15.17 (1.94)	15.38 (2.27)	15.15 (2.27)	0.84	
Weight z-score ^e^		196	-0.26 (1.12)	-0.21 (1.04)	-0.31 (1.19)	0.55	
Length z-score ^e^		196	-0.11 (1.40)	-0.08 (1.53)	-0.14 (1.53)	0.76	
BMI z-scores ^e^		195	-0.27 (1.33)	-0.24 (1.12)	-0.29 (1.50)	0.80	
BMI z-scores category ^f^		195				0.87	
Underweight and normal weight			175 (90%)	86 (90%)	89 (90%)		
Overweight			30 (17%)	8 (8%)	7 (7%)		
Obese			8 (5%)	2 (2%)	3 (3%)		
Sum of 4 skinfolds (mm)		190	34.29 (7.44)	34.06 (6.95)	34.50 (7.92)	0.56	
Timing of Assessments at one year							
Offspring age (months)		176	12.41 (0.94)	12.40 (1.01)	12.43 (0.88)	0.80	
Anthropometric Outcomes at one year							
Length (cm)		175	75.96 (3.40)	75.88 (3.43)	76.03 (3.41)	0.77	
Weight (kg)		175	9.78 (1.17)	9.82 (1.20)	9.75 (1.15)	0.69	
BMI (kg/m^2^)		175	16.93 (1.55)	17.03 (1.51)	16.85 (1.59)	0.44	
Weight z-score ^e^		175	0.27 (0.92)	0.30 (0.96)	0.25 (0.90)	0.77	
Length z-score ^e^		175	0.20 (1.21)	0.18 (1.24)	0.23 (1.19)	0.78	
BMI z-score ^e^		175	0.21 (1.09)	0.27 (1.10)	0.16 (1.08)	0.51	
BMI z-scores category ^f^		175				0.21	
Underweight and normal weight			137 (78%)	59 (74%)	89 (90%)	
Overweight			30 (17%)	18 (22%)	7 (7%)	
Obese			8 (5%)	3 (4%)	3 (3%)	
Sum of 4 skinfolds (mm)		163	37.95 (10.72)	37.68 (10.54)	38.16 (10.92)	0.78	
Fat free Mass (BIA) (kg) ^g^		163	8.42 (1.02)	8.40 (0.99)	8.44 (1.05)	0.84	
Total fat mass (BIA) (kg) ^g^		163	1.59 (0.75)	1.40 (0.68)	1.34 (0.80)	0.66	
Psychobehavioral outcomes at one year							
Parenting Stress Indicator – Difficult Child Subscale		152	22.38 (6.76)	22.39 (7.06)	22.38 (6.53)	0.99	
Total night time sleep duration (minutes)		160	639.36 (127.31)	648.31 (118.50)	631.36 (134.98)	0.43	
Number of night wakings		160	1.22 (1.23)	1.14 (1.14)	1.29 (1.30)	0.42	
Maternal Perception of child sleep as being “a very serious problem” (yes)		161	8 (5%)	3 (4%)	5 (6%)	0.62	

Continuous and normally distributed variables were described as means and standard deviations and ordinal outcomes were described as n (%). p-values were calculated via t-test for numeric outcomes and via Chi2 for ordinal outcomes.

Note; there were valid data for 178, 178 and 175 children at birth, 6-8 weeks and one year, respectively. This consisted of 84/105 (80%) offspring in the intervention and 95/106 (89.6%) offspring in the usual-care. All 179 offspring had less than 24% of missing data on all outcomes and this affected mainly the psychobehavioral outcomes. For the other outcomes, missing information was never higher than 9%.

BIA: Bioimpedance

BMI: body mass index

GA: Gestational Age

a Hyperbilirubinemia was considered as present if the neonate had total serum bilirubin levels equal or above 15 mg/dL (257 μmol/L) (55)

b Hypoglycemia was defined as capillary or venous glucose value ≤2.5 mmol/l (55) and preterm delivery was defined as birth delivery <37 GA.

c LGA: birth weight > 90^th^ percentile for sex and gestational age using the Intergrowth 21^st^ newborn size application tool

d SGA: birth weight < 10^th^ percentile for sex and gestational age using the Intergrowth 21^st^ newborn size application tool

e Calculated with the World Health Organization (WHO) child growth standards software (56).

f The WHO international growth references were used to calculate underweight, normal weight, overweight and obesity from z-scores, we used underweight: lower than −2 SD, normal: from −2 SD to < 1 SD, overweight: 1 SD to <2 SD, obesity from 2 SD and above.

g Butte formula, 2000: Fat free mass (kg)= Total Body Weight/age and sex specific hydration coefficient (79.3 for Boys or 78.8 for Girls) and Total fat mass = Mass – Fat free mass (62)

### 2.8 Statistical methods

All statistical analyses were performed with R (65). Descriptive statistics were computed as either number of individuals, means, and standard deviation or as the number of individuals and percentages (%) (**Tables 1** and **2**). In **Table 3**, we used linear regressions to evaluate between-group differences (usual-care versus intervention) in the offspring’s anthropometric and psychobehavioral outcomes. We used logistic regressions when evaluating between-group differences in the rates of the offspring’s adverse birth and neonatal outcomes (all), anthropometric binary outcomes, such as presence of macrosomia, SGA, LGA, and maternal perception of the offspring’s sleep as being “a very serious problem”. Finally, we used ordinal logistic regressions to assess between-group differences in the offspring’s BMI z-score category (being under or normal weight versus overweight or obese). We did not correct for multiple testing in any of the analyses due to the multifactorial nature of the intervention. No data was imputed. In the first basic model we did not adjust for any covariates, except for maternal age at first appointment as there was a significant difference between groups for this variable. In the second model, we additionally adjusted for offspring sex and age at the timing of the measure, where appropriate. When these covariates were not appropriate (e.g. no adjustment for age for the outcome: prematurity), we made note of it in the legend of the tables. We also tested, as covariates, breastfeeding at 6-8 weeks (yes/no) for outcomes at 6-8 weeks and breastfeeding at 6-8 weeks (yes/no) and 1 year for outcomes at 1 year. As these analyses revealed no differences in comparison to analyses made without these covariates we removed these covariates and kept our simpler model. A *post hoc* power analysis was conducted to see whether or not there was sufficient power to detect differences between the usual-care and intervention groups for our most important outcome: offspring weight. The *post hoc* power analysis revealed a power of 1, 0.99 and 1 to detect differences between groups for weight at birth, 6-8 weeks, and one year respectively, when using model 2. All statistical significance tests were accepted at p<0.05.

**Table 3:** Offspring’s between-group differences in birth, anthropometric, and psychobehavioral outcomes.

	Model 1		Model 2	
	*OR (95% CI)*	*p-value*	*OR (95% CI)*	*p-value*
Adverse Birth and neonatal outcomes				
Neonatal Hospitalization (yes/no)	1.14 (0.3, 4.4)	0.85	1.24 (0.28, 5.41)	0.78
Hyperbilirubinemia (yes/no)	0.36 (0.04, 3.61)	0.39	0.50 (0.05, 5.52)	0.57
Hypoglycaemia (yes/no)	0.75 (0.1, 5.92)	0.79	1.00 (0.11, 9.23)	.1.00
Prematurity <37 GA (yes/no)^a^	0.39 (0.13, 1.2)	0.10	0.38 (0.12, 1.17)	0.09
Anthropometric Outcomes	*β (95% CI) and* *OR (95% CI)*	*p-value*	*β (95% CI) and* *OR (95% CI)*	*p-value*
At birth				
Length (cm)	-0.22 (-0.11, 0.05)	0.56	-0.64 (-1.27, -0.01)	0.05
Weight (kg)	0.02 (-0.13, 0.16)	0.83	-0.06 (-0.19, 0.07)	0.34
Macrosomia (>4000 g) (yes/no)	1.58 (0.57, 4.39)	0.38	1.24 (0.43, 3.55)	0.69
Small for gestational age (yes/no) ^a,b^	1.17 (0.48, 2.81)	0.73	Not Applicable	Not Applicable
Large for gestational age (yes/no) ^a,b^	0.85 (0.35, 2.1)	0.73	Not Applicable	Not Applicable
Weight z-score^a,b^	-0.16 (-0.46, 0.13)	0.28	Not Applicable	Not Applicable
At 6-8 weeks				
Length (cm)	0.17 (-0.68, 1.01)	0.70	0.17 (-0.65, 1.00)	0.68
Weight (kg)	0.09 (-0.12, 0.29)	0.41	0.08 (-0.12, 0.28)	0.41
Weight z-score^a,b^	0.11 (-0.21, 0.42)	0.51	Not Applicable	Not Applicable
Length z-score^a,b^	0.05 (-0.35, 0.45)	0.81	Not Applicable	Not Applicable
BMI (kg/m^2^)	0.11 (-0.45, 0.67)	0.70	0.10 (-0.46, 0.65)	0.73
BMI z-score^a,b^	0.07 (-0.31, 0.45)	0.71	Not Applicable	Not Applicable
BMI z-score categories^a,b^	1.02 (0.40, 2.61)	0.96	Not Applicable	Not Applicable
Sum of 4 skinfolds (mm)	-0.19 (-2.36, 1.98)	0.86	-0.07 (-2.249, 2.11)	0.95
At one year				
Length (cm)	-0.03 (-1.07, 1.02)	0.96	-0.14 (-1.07, 0.79)	0.77
Weight (kg)	0.13 (-0.24, 0.49)	0.50	0.08 (-0.25, 0.42)	0.62
Weight z-score^a,b^	0.07 (-0.22, 0.35)	0.65	Not Applicable	Not Applicable
Length z-score^a,b^	-0.05 (-0.42, 0.33)	0.80	Not Applicable	Not Applicable
BMI (kg/m^2^)	0.22 (-0.25, 0.70)	0.35	0.20 (-0.28, 0.68)	0.41
BMI z-score^a,b^	0.14 (-0.20, 0.47)	0.42	Not Applicable	Not Applicable
BMI z-score categories^a,b^	1.56 (0.76, 3.23)	0.23	Not Applicable	Not Applicable
Sum of 4 skinfolds (mm)	-0.50 (-3.97, 2.96)	0.78	-0.85 (-4.26, 2.57)	0.63
Fat free Mass calculated (BIA) (kg)	0.00 (-0.32, 0.33)	0.98	0.00 (-0.31, 0.31)	0.99
Total fat mass (BIA) (kg)	0.07 (-0.17, 0.31)	0.58	0.05 (-0.18, 0.29)	0.66
Psychobehavioral outcomes at one year	*β (95% CI) and* *OR (95% CI)*	*p-value*	*β (95% CI) and* *OR (95% CI)*	*p-value*
Parenting Stress Indicator – Difficult Child Subscale	0.10 (-2.15, 2.34)	0.93	0.05 (-2.21, 2.32)	0.96
Total night time sleep duration (minutes)	20.58 (-23.29, 64.44)	0.36	27.1 (-17.67, 71.87)	0.23
Number of night wakings	-0.27 (-0.66, 0.12)	0.17	-0.31 (-0.72, 0.09)	0.12
Maternal Perception of child sleep as being “a very serious problem” (yes)	0.07 (-0.11, 0.26)	0.43	0.11 (-0.08, 0.30)	0.24

Effect estimates are based on the differences between the intervention and usual-care groups with p-values calculated from linear or logistic regressions.

Model 1 was adjusted to maternal age.

Model 2 was adjusted for the offspring’s age at the given time-point and his/her sex with the exception of outcomes that have an a or b (see below).

a No adjustment for the offspring’s age at that timepoint and for this outcome.

b No adjustment for the offspring’s sex at that timepoint and for this outcome.

BIA: Bioimpedance

BMI: Body Mass Index, see **Table 1** or methods for precisions on how these were calculated according to age and sex.

## 3 Results

Baseline maternal characteristics were similar between both groups (**Table 1**), except for maternal age, which was higher in the intervention group (34.48 and 32.79 years in the intervention and usual-care groups, respectively). Baseline offspring characteristics show there were no between-group differences in infant sex or age at the different time points (**Table 2**).

### 3.1 Adverse birth and neonatal outcomes

The rate of adverse perinatal outcomes was 5% for neonatal hospitalizations, 2% for hyperbilirubinemia and hypoglycemia and 8% for prematurity for all offspring together (**Table 2**). There were no between-group differences in the rate of neonatal hospitalizations, hyperbilirubinemia or hypoglycemia occurrences (all p ≥0.05, **Table 3**). Compared to usual-care, the rate of prematurity tended to be lower in the intervention group (p=0.10 in model 1 and 0.09 in model 2).

### 3.2 Anthropometric outcomes

The prevalence of LGA and SGA in the entire population were both 11%. The weight- and BMI z-scores were close to 0 in both the intervention and usual-care group at birth, 6-8 weeks and 1 year (**Table 2**). There were no between-group differences in the offspring’s anthropometry at birth, 6-8 weeks or one year (all p≥0.41, **Table 3**). Specifically, there were no differences in the occurrence of SGA and LGA, at birth, and no differences in weight, BMI, BMI z-scores or measures of adiposity (sum of four skinfold and/or BIA) at birth, 6-8 weeks or one year, except for birth length, which was lower in the intervention group compared to the usual-care group, but only in the adjusted analyses did it reach borderline significance levels (p=0.05).

### 3.3 Psychobehavioral outcomes

In the overall offspring population, the mean Parenting Stress Index was 22.38, the mean night-time sleep duration was 639 minutes/night with a mean of 1.22 awakenings/night and 5% of mothers perceived their child’s sleep as “a very serious problem” (**Table 2**). There were no significant between-group differences in the offspring’s psychobehavioral outcomes (all p ≥0.24, **Table 3**). Specifically, there were no differences in maternal perception of difficulty with self-regulation (as measured by the Parenting Stress Index), total nighttime sleep duration, number of night wakings and in the rates of maternal perception of the child’s sleep.

## 4 Discussion

To our knowledge, the *MySweetheart trial* is the first randomized multidimensional lifestyle and psychosocial intervention trial during and after pregnancy in women with GDM also intervening on the offspring up to one year. The intervention did not lead to a significant improvement in most birth and neonatal outcomes, offspring anthropometry, or their psychobehavioral outcomes up to one year compared to an active lifestyle and guidelines-based usual-care comparator. However, adverse birth and neonatal outcomes, increased anthropometric measures (weight, BMI and body fat), and psychobehavioral problems were very low in both groups. No between group differences were found for birth and neonatal outcomes. No between-group differences were observed in weight, BMI, BMI z-scores, sum of four skinfold, fat mass (BIA) at birth, 6-8 weeks or one year, except for birth length, which was -0.64 cm lower in the intervention compared to the usual-care group and was close to reaching statistical significance. Furthermore, there were no differences in maternal perception of self-regulation difficulty, the offspring’s total nighttime sleep duration, number of night waking, or the maternal perception of their child’s sleep as being “a very serious problem”.

Regarding birth and neonatal outcomes, we had very low rates of neonatal hospitalization, hypoglycemia, hyperbilirubinemia, and prematurity in both groups. Prior intervention studies investigating the effect of lifestyle interventions on offspring birth and neonatal outcomes are scarce. A recent retrospective analysis of a cohort using a special program for women, infants, and children (“WIC” program including nutrition education, breastfeeding education and support, referral to prenatal and pediatric health care and other maternal, child health, and human service programs) in women recently diagnosed with GDM showed a reduction in the risk of prematurity in the group of women with GDM who had benefitted from this program (25). This last finding is partially in line with our results, as we did find that the treatment effect on prematurity was close to being significant.

Anthropometric outcomes at birth were close to a healthy control population in the entire GDM group with 11% LGA and 11% SGA (defined as > 90^th^ and < 10^th^ percentile, respectively in an international control population (57). The cause for the reduced length of a mean of 0.64 cm found in model 2 for our intervention group is not entirely clear and has not been previously reported. Potentially, stunting could play a role, but the prevalence of SGA was not increased in the intervention compared to the usual-care group. One trial (n=932) that focused on adherence to Mediterranean diet in women with GDM found higher birthweight percentiles in the intervention group, but similar length percentiles compared to the usual-care group (66). Another intervention study conducted in women with GDM, which focused on chrono-nutrition and sleep hygiene from the time of GDM diagnosis until delivery, showed no differences in offspring anthropometry, particularly LGA, compared to the usual-care group (67). The mean BMI Z-scores of the entire GDM population were close to a healthy control population of the WHO (-0.27 and 0.21 at 6-8 weeks and 1 year) with only 5% being obese (13). Thereby, we did not find any between-group differences in any of the anthropometric outcomes at these two time points. Comparing anthropometric outcomes to other studies beyond birth, a review concluded that treating obese pregnant women through lifestyle interventions consisting of diet and/or physical activity had a limited impact on offspring anthropometry during childhood (26). Other studies even showed an adverse impact of interventions on the offspring’s anthropometry. For instance, the above-mentioned retrospective analysis of the “WIC program” reported that the cohort of women with GDM in the program gave birth to larger offspring than the women not participating (25). All these studies show that intervening on maternal lifestyle behavior has a limited impact on the offspring’s anthropometry.

Regarding psychobehavioral outcomes in the offspring, the fact that we used a subscale of the Parenting Stress Index makes it difficult to compare to prior studies. However, regarding sleep, the mean night-time sleep duration was 639 minutes/night, which is lower than the Swiss mean recommended amount per night, as described in a previous paper in women with GDM (18). Our mean of 1.22 night wakings is similar to prior literature in the general population at the same age (68, 69) and the prevalence of 5% of mothers perceiving their child’s sleep as “a very serious problem” is relatively low. Another systematic review and meta-analysis in the general population demonstrated that difficulty in the offspring’s behavior and self-regulation may be reduced by any type of parenting intervention taking place from pregnancy and up to three years, as these interventions promote infant psychobehavioral development and reduce behavior problems (70).

In general, our results align with prior studies performed in other populations with and without GDM. Importantly, we had a very active guidelines-based control group. Thus, in both groups, the rates of perinatal adverse events were very low and anthropometric outcomes were close to a healthy control group despite women having GDM. Similarly, the number of night wakings were comparable to the general population. Also, rates of breastfeeding were 96% in the intervention and 97% in the usual-care group at 6-8 weeks and 71% in the intervention and 72% in the usual-care group at one year, which is much higher than in the general Swiss population. The fact that the offspring of this study had better outcomes, or outcomes comparable to the general swiss population, and the fact that there were no major improvements regarding outcomes in the offspring from the intervention group suggests that our usual care already follows high standards and allows improvements in offspring outcomes. This absence in differences could also be explained with participation bias, whereby individuals agreeing to take part in the study are individuals who may be more willing to make positive changes in their lifestyle behaviors. Finally, it could be explained by the fact that clinicians seeing these women were aware that a study was taking place and may have delivered higher quality care to women from both groups.

An interesting avenue for future studies seems to be the use of technology rather than face-to-face interventions focusing specifically on infant nutrition and health. As described above by our intervention did use text messaging to summarize behavioral goals for the mother, but less focus was placed on the offspring’s behavioral goals, as our primary aim was to reduce maternal weight and depression symptoms. Thus, the lack of group differences in our study may have been caused by the larger focus placed on maternal outcomes and may have involved too many components and behaviors to adapt in a short period. We suggest that future interventions should focus on technological interventions that target behaviours in the offspring beyond the first year of life, given their higher risk of childhood obesity (8, 71). This is especially challenging in a more high-risk and vulnerable population. Furthermore, once the offspring was born, face-to-face interventions were less frequent, and the mothers less available. Therefore, mother and child were followed up mostly by phone, which could have led to a lower adherence regarding goals for the offspring. Also, breastfeeding rates in both groups were very high and similar, which probably contributed to the favorable anthropometric and psychobehavioral outcomes observed in the offspring of both groups.

## 5 Strengths and limitations

This complex lifestyle and psychosocial intervention focusing on many important factors and intervening both on the mother and the offspring in a relatively large sample is an important strength of this study. The limitations of this study first concern the fact that we had an active lifestyle and guidelines-based usual-care group that also received important advice about maternal lifestyle behaviors and breastfeeding, limiting potential intervention effects. This differs compared to other populations such as healthy control or obese women, as women with GDM are regularly followed up and current recommendations also include postpartum visits. Furthermore, the intervention group may have had too many maternal and infant health goals to be able to implement them. We did not measure adherence to the behavioral goals regarding the offspring, such as timing of introduction of solid foods or objective measures of physical activity (accelerometry), as this would have increased the participant burden. However, the mothers were regularly followed up by the coach who reviewed the adherence with the participants. Moreover, the WHO recommends exclusive breastfeeding for at least 6 months. However, in accordance with the local pediatricians and the usual practice in Switzerland, we based ourselves on European and Swiss guidelines to introduce solid foods after at least 4 months of age. This could be considered as a potential limitation. Unfortunately, we did not formally check if women were still breastfeeding at 6 months. In addition, the psychobehavioral outcomes were solely assessed *via* maternal self-report. As the primary power analyses focused on maternal outcomes, conclusions need to be drawn tentatively and replication of results would be needed.

## 6 Conclusion

Knowledge regarding the importance of the transgenerational impact of metabolic diseases has increased in recent years. This is the first interdisciplinary lifestyle and psychosocial pre- and postpartum intervention in women with GDM that also focused on their offspring. It did not lead to a significant improvement in most birth and neonatal outcomes, offspring anthropometry or psychobehavioral outcomes in the offspring up to one year compared to an active lifestyle guidelines-based usual-care group, but the rates of adverse outcomes were very low in both groups and anthropometric and psychobehavioral outcomes were similar to healthy control populations. Prematurity tended to be lower in the intervention group and length at birth was reduced in the intervention group, although, these between-group difference were only close to reaching statistical significance. Also, the clinical significance of the reduction in length at birth remains to be elucidated. There were few adverse outcomes in both groups and no differences between the intervention and the guidelines-and lifestyle-based usual-care group. Thus, we could conclude that following the current guidelines in mental health and lifestyle recommendations in women with GDM stringently, following up and motivating patients regularly might be sufficient, although this should continue far beyond one year postpartum. A future trial to improve offspring outcomes in mothers with GDM might benefit from an intervention starting early in pregnancy, a stronger focus on the offspring behavior and health, as well as on novel, culturally adapted technologies and should continue far beyond one year postpartum.

## Data availability statement

The raw data supporting the conclusions of this article will be made available by the authors, without undue reservation.

## Ethics statement

The studies involving human participants were reviewed and approved by Human Research Ethics Committee of the Canton de Vaud (study number 2016-00745). Written informed consent to participate in this study was provided by the participants’ legal guardian/next of kin.

## Author contributions

JP and AH conceived the study, designed the trial, obtained grant funding, and oversaw management of the trial. LG, DQ, JG and SL helped in designing parts of the study and participated in the implementation of the study. LG performed the data analysis with the help of AL, BS and DQ. All authors (LG, DQ, AA, AH, and JP) were involved in the interpretation of data. LG and DQ wrote the draft manuscript. Authors LG, DQ, SS, JG, AA, AH, AL, SL, BS and JP revised the manuscript for important intellectual content and gave final approval for the version to be published. JP and AH supervised all the work. All authors contributed to the article and approved the submitted version.
